# Analysis of snakebite data in Volta and Oti Regions, Ghana, 2019

**DOI:** 10.11604/pamj.2021.40.131.28217

**Published:** 2021-11-03

**Authors:** Baba Ceesay, Abdoulie Taal, Momodou Kalisa, Magdalene Akos Odikro, David Agbope, Ernest Kenu

**Affiliations:** 1 Ghana Field Epidemiology and Laboratory Training Programme (GFELTP), School of Public Health, University of Ghana, Accra, Ghana,; 2 Ministry of Health, Banjul, The Gambia,; 3 Volta Regional Health Directorate, Ho, Ghana

**Keywords:** Snakebites, incidence, rain, disease outbreaks

## Abstract

**Introduction:**

globally about 5.4 million people are affected by snakebite annually leading to 2.7 million cases of snakebite envenoming and 81,000-138,000 deaths. In sub-Saharan Africa, the burden of disease caused by snakebite is often underestimated despite its status as a category A neglected tropical disease. We reviewed snakebite data to determine the magnitude of snakebite by person, place, and time in the Volta and Oti Regions of Ghana.

**Methods:**

we conducted a descriptive secondary data analysis using snakebite data from 2014-2018 extracted from the District Health Information and Management Systems (DHIMS 2) database. Data were analyzed descriptively by person, place, and time using summary statistics and results were presented in proportions and graphs. Missed outbreaks were determined through calculation of cumulative sum (CUSUM 2).

**Results:**

a total of 2,973 cases of snakebites were reported over the 5 years of which 1675 (56.3%) were males. Majority 867 (29.2%) of snakebite victims were between 20-34 years of age with recorded 5-year average incidence of 24 snakebite cases per 100,000. Nkwanta North District recorded the highest cases 499 (16.8%) with most of the snakebite cases 2,411 (81%) recorded in the rainy season. Overall, there was a decreasing trend of snakebites and four missed snakebite outbreaks occurred during the period. No snakebite death was recorded.

**Conclusion:**

a 5-year average snakebite incidence of 24 cases per 100,000 persons was recorded and Nkwanta North District recorded the highest cases with peaks occurring in rainy and harvesting seasons. Four outbreaks were missed. There is a need to conduct periodic data analysis for effective intervention programs.

## Introduction

Globally about 5.4 million people are affected by snakebite every year which leads to 2.7 million cases of snakebite envenoming and 81,000-138,000 deaths annually [1]. Worldwide, an estimated 7,400 people are bitten by a snake daily leading to the death of 220-380 people each day [2]. About 3-5.5% of victims of snakebite suffer from amputation and complications caused by envenomation [3], which may lead to permanent physical damage due to tissue necrosis, persistent nerve damage, and spat venom-ophthalmia [4,5]. The actual burden of snakebites is grossly underestimated due to the fragile reporting systems and management of snakebites outside hospital settings therefore the impact of snakebite is not known in many parts of the world [6]. With requests and advocacy from stakeholders on the unaddressed burden of snakebite envenomation in healthcare development efforts despite increasing incidence, severity and clinical characteristics, snakebite envenoming was formally included as a category A Neglected Tropical Disease (NTD) by the World Health Organization (WHO) in June 2017 [7-9].

In sub-Saharan Africa, the burden of disease caused by snakebite is often commonly underestimated [5]. The region reports a projected snakebite envenomation of 10,001 to 100,000 leading to an estimated 1,001 to 10,000 deaths per year [4]. Overall, 95% of snakebite cases occur in tropical and low-income countries [9]. The majority of bites occur among people with low socioeconomic status and have been linked to the occupations such as farming, fishing, animal rearing, and hunting [1,7,10,11]. Additionally, significant proportion of victims of snakebites are young people causing increasing economic impact due to loss of workforce [5,12].

In Ghana, the common snake species that causes envonomation is the saw-scalled viper Eschi Ocellatus [13,14]. A study in Western Ghana reported snakebite incidence of was 82.8 per 100,000 with Bole and Kintampo recording incidence of 110/100,000 and 74/100,000 populations respectively [4,15].

As part of the integrated disease surveillance and response reporting, snakebite data are routinely collected from the community and sub-regional level and transmitted to the regional level. However, beyond the collation and aggregation of the data at the regional level, the snakebite data are not adequately monitored leading to limited knowledge about the magnitude, spatial distribution, and possible undetected outbreaks of snakebite in the regions. Analyzing snakebite data in the districts will inform the regional health directorate about the pattern of snakebite in the regions and inform targeting of appropriate preventive and control measures. We analyzed routinely collected snakebite data to determine its magnitude and distribution by person, place, and time over a 5 year period in the Volta and Oti Regions of Ghana.

## Methods

**Study design:** we conducted a descriptive secondary data analysis of reported snakebite cases in the Volta and Oti Regions of Ghana. Data on snakebite data for the period 2014 to 2018 was extracted from the District Health Management and Information System (DHMIS 2) database.

**Study setting:** the study was carried out in the Volta and Oti Regions, which are two of Ghana´s sixteen administrative regions. The previous Volta Region was recently divided into two regions namely Volta and Oti Regions. The Oti and Volta Regions are located in the eastern part of the country sharing boundaries in the north with the northern region, the south with the Gulf of Guinea, in the west with the Volta Lake, and east with the Republic of Togo. Both regions are a blend of undulating highlands and lowlands with green vegetation and a 359 km^2^park contains both forest and savanna species of plants and animals.

The main activities in the regions are agriculture, hunting, forestry, and fishing [16]. Together, the regions occupy a surface land area of about 20,570 kilometers^2^representing 8.6% of the total landmass of Ghana. Recently the region has been. There are 692 health facilities across the two regions, which are made up of 29 hospitals, 153 health centers, 43 clinics, 441 functional community health planning and services (CHPS), and 14 maternity homes. The regional health information unit is responsible for the management of health data and health facilities report snakebite cases monthly through the integrated reporting system. As at the time of this study, the health administration of the regions was yet to be divided.

**Data collection and analysis:** we extracted 2014-2018 snakebite data from the DHIMS 2. DHIMS2 is an electronic integrated database that contains a variety of diseases that includes snakebites. Variables that were collected included the number of cases reported, age, sex, and reporting districts of the cases.

We exported the data into Microsoft Excel 2013 for cleaning and analysis. Data were analysed descriptively by person, place and time using frequencies, proportions, ratios and maps. Categorical data were presented using summary statistics, tables and charts and continuous variables using summary statistics. Additionally, we drew Geographical Information System (QGIS) software maps to show the distribution of cases by place. In calculating the incidence of snakebite, the total number of snakebite recorded per year was divided by the mid year population. The overall average snakebite incidence was calculated by using the five year average mid-year population and recorded snakebite cases. All incidences were reported per 100,000 population. We used the C2 Cumulative Sum (CUSUM) to determine any missed outbreaks that may have occurred over the period. The C2 which represented the baseline number of cases was calculated by using the formula; C2 = mean + 3* standard deviations of 7 past surveillance points before a 2 months lag [17]. The number of snakebite cases reported was then compared to the number expected to be reported in the particular time frame to detect any increase that represents an outbreak.

**Ethical issues:** since snakebite is reported as part of the Integrated Disease Surveillance and Response (IDSR), and de-identified secondary data was used, no formal ethical approval was required. The Ghana Field Epidemiology and Laboratory Training Programme of the University of Ghana sought permission from Ghana Health Service through the Regional Director of Health Services, Volta Region to conduct data analysis.

## Results

A total of 2,973 cases of snakebites were reported in the Volta and Oti Regions for the period under study. Out of the total cases recorded 56.3% (1675/2973) were males. Most of the cases 82% (2411/ 2973) were registered in the rainy season (Table 1).

**Table 1:** characteristics of snakebite cases, Volta and Oti Regions, 2014-2018

Variable	Frequency	Percent
**Sex**		
Male	1675	56.3
Female	1298	43.7
**Age**		
<1	16	0.5
1-4	99	3.3
5-9	186	6.3
10-14	274	9.2
15-17	228	7.6
18-19	232	7.8
20-34	867	29.1
35-49	549	18.4
50-59	260	8.7
60-69	136	4.5
70+	126	4.2
**Region**		
Volta	1163	39.1
Oti	1810	60.9
**Season**		
Dry	562	18.9
Rainy	2411	81.1

**Figure 1:**
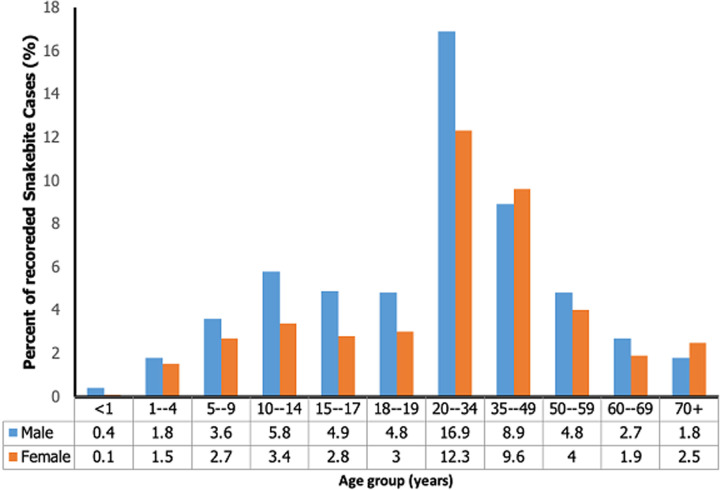
age and sex distribution of snakebite cases, Volta and Oti Regions, 2014 to 2018

There has been a reduction in the incidence of snakebite in the region over the period. The incidence was higher in 2014 with 32 cases per 100,000 population and the lowest incidence of 19 cases per 100,000 population in 2018. However, in 2016, there was a slight increase in the incidence of snakebite with 25 cases per 100,000 population (Table 2). The highest proportion of cases 29.2% (867/2973) was recorded between the age group 20-34 years and the least was less than 1 year of age (0.50%). The proportion of snakebite cases by age category was also higher among males for all age categories except for the age group 35-39 years and above 70 years. However, snakebite cases occurred in both males and females of all ages in the region (Figure 1).

**Table 2:** incidence of snakebite, Volta and Oti Regions, 2014 to 2018

Year	Mid-year population	No of cases	Incidence/100,000
2014	2338151	751	32
2015	2396397	563	23.5
2016	2456262	607	25
2017	2517626	551	22
2018	2580523	501	19
Overall average	2457791.8	594.6	24

**Figure 2:**
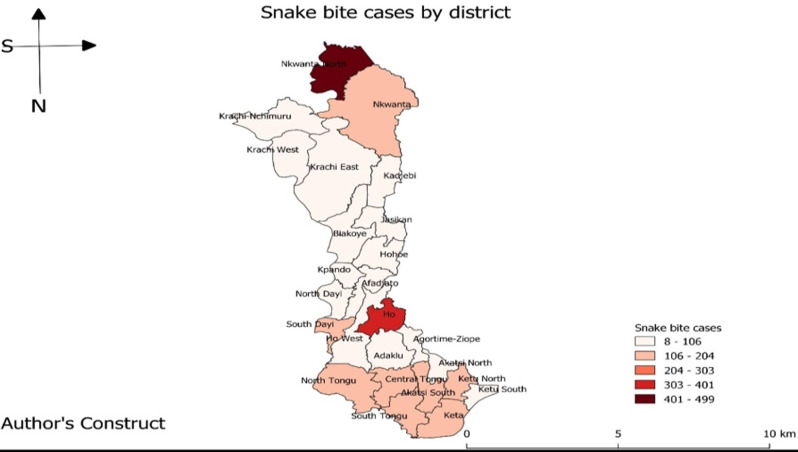
snakebite cases by district, Volta and Oti Regions, 2014 to 2018

**Figure 3:**
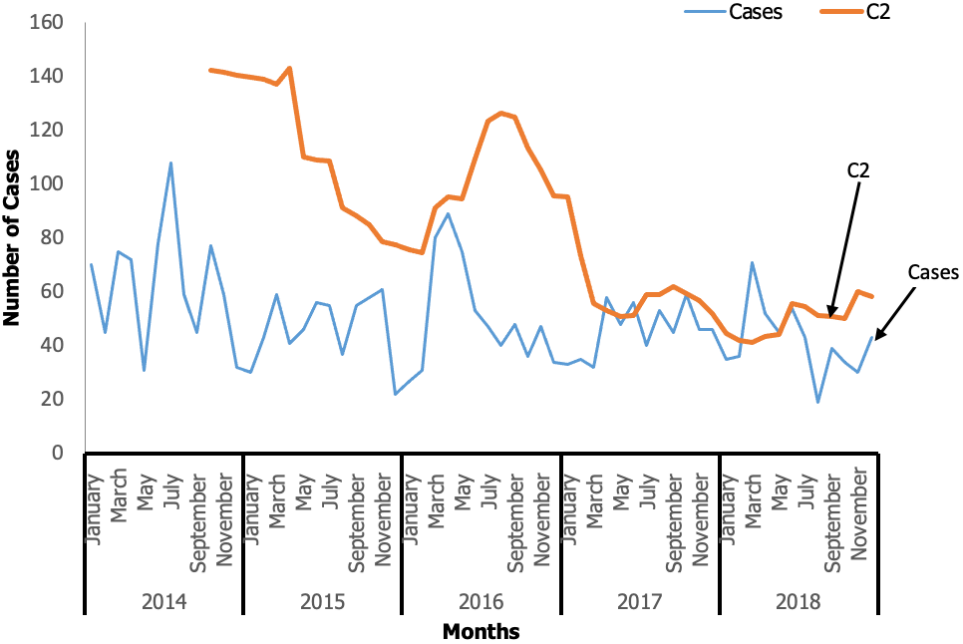
snakebite cases by month and threshold (C2), Volta and Oti Regions, 2014 to 2018

No snakebite death was recorded. Nkwanta North District recorded the highest number of cases 16.8% (499/2973), followed by Ho District 11.8% (352/2973), and the lowest cases were recorded in the Akatsi North District 0.26% (8/2973) (Figure 2). Using the C2 CUSUM, from 2014 to 2018 four snakebite outbreaks occurred in the region. These outbreaks were missed by the health officials over the period. The outbreaks occurred in 2017 (April and June) and 2018 (March and April) (Figure 3).

## Discussion

From 2014 through 2018, there has been a decrease in the incidence of snakebite cases in the Volta and Oti Regions. Overall, the average incidence of snakebite recorded in this study was 24 snakebite cases per 100,000 populations. This is relatively lower compared to a study in the Amazon, Brazil which reported an incidence rate of 52.8 cases per 100,000 people per year and another study in Ghana that reported incidence of 92 snakebites cases per 100,000 population [4,18]. The study in Northern Ghana used a prospective design which meant that the chances of finding snakebite cases will be higher than using routine reporting process as was done in our study. Additionally, studies in sub-Saharan Africa have reported incidence of snakebite that ranges between 150-250 snakebite cases per 100,000 population and few regions in Nigeria have an incidence of 497/100,000 population [19]. The incidence recorded in our study is however, likely to be underestimated as pertains to settings in Ghana and sub-Saharan Africa. The majority (50-90%) of snakebite victims in sub-Saharan Africa visit traditional healers as a first-line treatment instead of health centers and this contributes to underestimation of snakebite incidence [5,9].

We found that both males and females were affected by snakebite but the majority (56.3%) of the cases were males. This is in line with two studies conducted in the Northern Region of Ghana which showed similar results with 67.7% and 70% of the snakebite victims respectively being males [10,20]. The nature of activities by men in rural areas may be the possible contributing factor to the high number of snakebites which includes hunting, fishing and farming. Snakebite was recorded among all age groups in the region. The highest number of cases was recorded between the age group 20-34 years, who are the economically productive age group. Our finding is similar to other studies that reported that adult populations were most at risk of snakebite [18]. Mensah *et al*., 2016, in their study in the Western Region of Ghana also found that snakebite was common among the age group 20-34 years [15].

Our analysis also showed that the northern part of the region reported the highest number of cases. Nkwanta North District is a typical rural setting which is located in the north of the region and recorded the highest proportion of cases (16.8%). The presence of the rich vegetation and the farming activities all year round could have contributed to the high number of snakebites in the district. This conforms to two different studies in Brazil and India which reported that about (68.6%) and (86.9%) of all the snakebites occurred in the rural parts of the country [21]. Other studies suggest that rural people are mostly at risk of snakebite due to their daily routine activities and intrusion of habitats through agricultural activities increase their exposure [22]. Salve *et al*. in their study showed that agricultural activities and favorable geographic location have been linked to the high incidence of snakebite in most districts in India [1]. The second-highest number of cases during the period was recorded in Ho Municipality. The municipality has a teaching hospital, which is the main referral center in the region. The high number of cases could be attributed to the cases referred from peripheral health facilities in the region.

Our findings revealed that the peak of snakebite cases occurred during the rainy season and harvesting period between March to July and August to November. Invariably, the seasonal variation continues to support the growing number of snakebite cases in most parts of the world noticeably in India where the majority of snakebites occurred in the monsoon season (May to November) [1]. This is similar to a retrospective study in Northern Ghana and another study conducted at the Tamale Teaching Hospital in Ghana that reported the highest number of snakebites in April (14.3%), June (12.4%), and November (12.4%) in Northern Ghana and in November (10.3%) in the Tamale Teaching Hospital [4,23]. Additionally, in a study conducted in Andhra Pradesh, India, authors reported that the rainy season provides habitation for snakes and direct contact with farmers [22]. The chances of having snakebites during these periods are high as it is the peak period for farmers in Ghana to engage in farming activities leading to the risk of snakebites.

**Limitations:** the limitation of this study was the inadequate information on characteristics collected about the snakebite cases. The DHMIS 2 did not capture the type of snake, site of the bite, treatment, and occupation of the cases reported. Our findings may not be the true reflection of the actual incidence of snakebite in the region because only those who reported to the clinic were considered. Additionally, we also use the mid-year population of the region as the denominator to estimate the incidence of snakebite therefore our reported incidence should be considered in light of this.

## Conclusion

Over the five year period, a total of 2,973 snakebite cases were recorded with 5-year average incidence of 24 cases per 100,000 population. Majority of the recorded snakebite cases were males and young people between the ages of 20-34 were most affected. Nkwanta North District registered the highest number of snakebite cases. Rainy and harvesting seasons were the peaks of snakebites with a recorded decreasing incidence across the years in the regions. Four snakebite outbreaks were missed over the period. Regional and health facilities staff need to increase awareness on snakebite in communities and conduct periodic data analysis to know the distribution of snakebites. Improvement on the variables on snakebite in the DHIMS 2 by the Ministry of Health (MoH) will enhance adequate data analysis for planning and intervention purposes.

### What is known about this topic

- Snakebite is a neglected tropical disease;
- Rural people and agricultural workers are mostly affected.

### What this study adds

- Understanding the annual incidence of snakebite in the Volta and Oti Regions from 2014 to 2018;
- Age group 20-34 years were most affected with snakebite;
- Regular analysis of snakebite data is essential to prevent missed outbreaks.
